# LabOps: A flexible self-hosted workflow of open source tools for efficient collaboration within research laboratories

**DOI:** 10.1371/journal.pcbi.1013248

**Published:** 2025-07-01

**Authors:** Héctor D. García-Verdugo, Cristian Román-Palacios

**Affiliations:** College of Information Science, University of Arizona, Tucson, Arizona, United States of America; Montreal, CANADA

## Abstract

Effective collaboration, essential for success in academic environments, often requires efficient team communication and access to a team-oriented digital infrastructure. Despite the significance of efficient and effective collaboration within the academy, a standardized and structured suite for collaboration remains relatively overlooked. In the context of research labs, where multiple levels of collaboration often coexist, the ability to communicate and share resources in a timely and secure manner is critical. Modern research teams and institutions have historically attempted to solve these needs through proprietary, largely restrictive, and inflexible tools. Free and Open Source Software (FOSS) has facilitated the development of our modern computer-focused world by virtue of its legal, customizable, and accessible nature. However, FOSS remains weakly used for supporting communication, collaborative writing, storage, and other tasks within research labs. This paper discusses the implementation of a FOSS computational workflow for active collaboration within academic environments. We focus on identifying available tools that can support collaborative writing, instant messaging, data storage, among other tasks. The Lab Operations (LabOps) workflow presented in this paper—which can be self-hosted, adapted, and adopted at different levels—offers an alternative approach to off-the-shelf proprietary solutions for both within- and cross-lab communication. We discuss the benefits, flexibility, limitations, and potential approaches to adoption of the workflow.

## Author summary

Is there anything beyond Slack, Microsoft Teams, Google Drive, and OneDrive? Effective collaboration is crucial for success in academic environments, yet a standardized suite of open-source tools for structured collaboration remains underutilized. In this paper, we present a workflow composed of Free and Open Source Software (FOSS) that enables research labs to self-host tools for communication and storage with relative ease. Through this Lab Operations (LabOps) workflow, academics can enhance collaborative efficiency while strengthening data security and sovereignty over the long term. With the right tools, research labs can achieve levels of collaboration and accessibility comparable to commercial platforms—without the associated costs, access limitations, or privacy concerns. We also discuss the practical limitations of our workflow and outline strategies for effective adoption.

## Introduction to collaboration within academia

Collaboration has driven many of the most significant discoveries and advancements in human knowledge [1]. Specific elements crucial to successful collaboration—such as the number of collaborators, publications, grants, and students—are directly tied to overall performance within academic environments. These elements include efficient communication among personnel, students, and staff, strong project management skills (e.g., managing grants, projects, and papers), and effective time management [2–4]. Despite the undeniable importance of these quantitative indicators of success, traditional methods of collaboration within academia and industry often struggle to keep pace with the evolving landscape of how collaborations are initiated and maintained from social, academic, and technical perspectives [5,6]. The efficient exchange of information and ideas is essential for better utilizing resources, especially given that modern scientific collaborations are increasingly data-intensive and interdisciplinary.

Various tools and resources have been developed to improve how collaborations are conducted within academia, regardless of discipline or geographical location. While these platforms represent significant progress in enabling modern lab-based collaboration, they often fall short in providing the accessibility, flexibility, and customization required by diverse research environments. For instance, free tiers of some tools limit access to critical data—such as chat history—to a restricted time window. Similarly, while some systems allow for easy data storage, they are not user-friendly when exporting or migrating large datasets. Proprietary tools also frequently fail to uphold data sovereignty and security for personal and scientific information used by the broad range of institutions, labs, and researchers [6,7]. Software is undeniably a key element of modern scientific collaboration. As such, it must be examined in greater depth regarding how it both enables and constrains collaboration within academic settings [8].

## Free and Open Source Software (FOSS) and collaboration in academia

We focus on Free and Open Source Software (FOSS). FOSS is licensed under an Open Source Initiative (OSI) copyright license that makes source code freely available for access, use, editing, and redistribution. FOSS has played an influential role in software development and, consequently, in modern collaboration. Well-known examples of FOSS that have had a major impact on the user-computer sphere include GNU/Linux, the Apache HTTP server, Mozilla Firefox, Git, and MySQL, among others [9]. Open source programming language projects such as R and Python have helped industry and academia around the world simplify experimental pipelines, compile and analyze information, create graphical outputs, and apply robust statistical workflows to datasets [10]. Open source tools like BLAST, Bioconda for sourcing bioinformatics software, as well as a plethora of R packages and Python libraries, have significantly transformed how collaboration and research take place across many biological disciplines. These tools have built—and continue to build—the foundation for our current computing- and data-intensive world, fostering a more collaborative, innovative, and inclusive approach [11].

FOSS has its beginnings in the early days of computing [12]. The intentional development of open source software emerged in response to the commercialization of software in the 1970s [13,14]. The rise of commercially available software created a legal environment in which proprietary software could not be freely shared or improved. In contrast, proponents of open source software emphasized the importance of the freedom to redistribute, access, and edit code [13–15]. Today, many of the most widely used tools for collaboration in academic and industry settings are private and closed source [7]. A relevant example is the Microsoft Office suite (Microsoft 365), a set of tools that support document creation, editing, sharing, and more. However, FOSS development is often explicitly positioned as an optimal alternative—or even a direct counterpoint—to these proprietary tools. We suggest that the sustained emphasis on open, collaborative software development not only challenges the dominance of proprietary platforms but also promotes collaboration that is more structured, inclusive, and dynamic—ultimately reinforcing fundamental principles related to innovation and accessibility [16,17].

The modern landscape of collaboration and communication for scientists and academics has changed dramatically over the last couple of decades. Yet the overall framework has retained essential components for efficient research. From investigation once done primarily in libraries, and communication via postal mail and phone, to today’s use of online publication search engines like Google Scholar and group communication platforms like Slack, academics continue to refine collaborative methods for greater efficiency and speed. Research laboratories have adapted to using collaboration tools tailored to their needs. One such example is the concept of “collaboratories” or “centers without walls,” in which scientists can conduct research regardless of physical location—enabling communication, data sharing, information exchange, and computational collaboration [18]. Historical applications of collaboratories emphasized tool-centric (e.g., text chat, video conferencing), data-centric (methods of accessing data), and data-sharing–centric (methods for sharing data) approaches. However, they often lacked a focus on FOSS principles—resulting in workflows that were unstructured, difficult to share, and easily forgotten [19]. We propose that a modern collaboratory should implement the best features of FOSS in science: open source, accessible, sovereign, and capable of being customized, upgraded, and modularized for specific research needs. The pool of available FOSS tools is ever-changing—some software may begin as open source but later transition to different models, affecting availability or feature support for different user groups. The framework presented here is named Laboratory Operations (LabOps), a term inspired by operational practices in other fields (e.g., DevOps, MLOps).

## Self-hosting a FOSS workflow for collaboration

**Fig 1:**
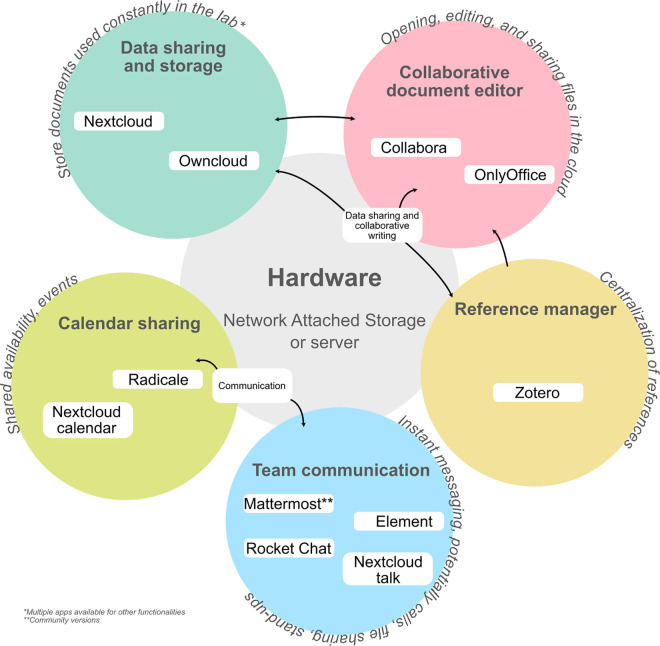
Diagram of the selfhosted LabOps workflow Free and Open Source Software (FOSS) in relation to hardware. Summary of the selected tools and resources used to deploy an integrated system for communication within the lab. We provide additional details in Table 1. We list (1) Nextcloud and ownCloud under Data sharing and storage, (2) Collabora and Onlyoffice under Collaborative document editor, (3) Nextcloud calendar and radicale under Calendar sharing, (4) Zotero for Reference manager, and (5) Mattermost, Element, Rocket.Chat, and Nextcloud chat for Team communication. In the figure, descriptions are particular to community editions. Central to the LabOps is the hardware configuration (could be a server, NAS, or less specialized hardware such a laptop, mini pc or similar).

We compiled a set of free and open-source software (FOSS) to self-host and either support or entirely replace specific tasks that are generally performed using traditional closed-source workflow environments. Our integrated workflow includes: (1) a dynamic project management and communication platform, (2) a document storage and sharing environment, (3) a suite of document creation and editing tools, (4) a task and calendar server, and (5) a reference citation manager. Our selection of tools was based on a thorough comparison of features across similar open-source and closed-source projects, followed by direct and extensive testing between August 2023 and June 2025. Although several of the FOSS tools discussed below are also available as commercial software due to development led by companies with commercial interests, community versions can still be downloaded, installed, and deployed at no cost (aside from hardware). However, we note that fluctuations between commercial and community versions often lead to the discontinuation of features as new versions are released. Readers should carefully review the latest version of each tool at the time of installation and evaluate their usefulness within the context of their LabOps workflow. We provide details on the LabOps workflow presented in this study in Fig 1 and Table 1.

**Table 1:** List of Free and Open Source Software (FOSS) used in the workflow. Composition of the open source collaboration focused workflow. A list and description of all the open source software that can be self hosted in a Network Attached Storage system (NAS) is presented and described below. We note, however, that Zotero is not being self-hosted for this solution but could self-hosted if needed. We also note that the panorama of functionality and FOSS access changes quickly in accordance to the development of some of these resources.

Name	Target task	Description	Notes	Link
OnlyOffice/Collabora	Document editor	Open source document editor that consists of a suite of editing and collaboration software that closely resembles Microsoft Office suites. OnlyOffice offers most of the service capabilities needed for traditional academia (e.g., document, presentation, spreadsheet, PDF editors). Live collaboration in the cloud is allowed.	We consider LibreOffice and Collabora Office as viable alternatives for collaboration on documents. Their integration with ownCloud and Nextcloud imply that files can be edited in the cloud and stored directly on the storage service.	OnlyOffice: https://www.onlyoffice.com/; Collabora: https://www.collaboraonline.com/
Nextcloud/ownCloud	Cloud collaboration system	Open source project built with content collaboration in mind. Documents and files in general can be created shared and synced. Resembles commercial solutions like Google Drive, Dropbox, and Microsoft OneDrive.	Both closely integrate with OnlyOffice (and other) for simultaneous collaboration and storage on the cloud. Nexcloud has a number of additional apps for communication, calendar sharing, among others. External users can view and edit files shared with them directly on the browser (using OnlyOffice). Registered users can be associated to particular roles and access to folders can be personalized in accordance with roles.	ownCloud: https://owncloud.com/; Nextcloud: https://nextcloud.com/
Mattermost/ Nextcloud talk	Instant messaging	Online chat service, project management platform with integrated file sharing. Resembles current and widely used group messaging communication platforms like Slack, Discord and Microsoft Teams.	A large number of plugins are available (e.g., RSS subscriptions, stand-ups, kanban boards). Access to features varies across versions. Viable alternatives are Element/Matrix, Nextcloud Talk, among others.	https://mattermost.com/
Zotero	Reference manager	Free and open-source reference management software to manage bibliographic data and related research materials. Zotero resembles popular citation managers like EndNote.	Integrates with OnlyOffice. Zotero is also self-hostable, although we currently use a cloud-version of the service. An alternative approach could utilize Jabref.	https://www.zotero.org/
Radicale	Calendar sharing	Free and open source, self-hostable, calendar and address book server. It uses and implements CalDAV standards. Strong replacement for classic closed-source clients and services such as Google Calendar and Microsoft Outlook Calendar.	Documentation is very limited. Can be used to bridge between the poor compatibility between Outlook and Google services. It can be replaced by Nextcloud Calendar or similar.	https://radicale.org/

### Software

In our workflow, Mattermost, Nextcloud/ownCloud, Radicale, and OnlyOffice were deployed using Docker images. Docker is an open-source platform that automates application management through containerization. Containers enable applications to run in any environment while minimizing compatibility issues, achieved by packaging the necessary dependencies, libraries, and configuration settings within a universal computing environment. We deployed and managed these tools using Portainer within our Network Attached Storage (NAS) system (see “Hardware and operating systems”). We provide ready-to-deploy and pre-configured.yaml files for Mattermost, Nextcloud/ownCloud, Radicale, and OnlyOffice in the GitHub repository associated with this paper (https://github.com/datadiversitylab/LabOps). However, either pre-configured or extensively annotated.yaml files for Docker Compose are also commonly available in relevant project repositories. In our LabOps workflow, Zotero was integrated using a plugin for OnlyOffice, with its database linked through a free Zotero account. Finally, we note that Nextcloud partially integrates functionalities from Mattermost (via Nextcloud Talk), Radicale (via Nextcloud Calendar), and ownCloud (via Nextcloud Files). This intentional redundancy in the workflow presented in this paper is advantageous to delineate an optimal strategy for adoption (see “Strategies for adoption”).

Two aspects are central to the deployment and proper functionality of these applications for collaboration. First, a predefined folder structure must be followed within the NAS to mount specific volumes to containers. This structure ensures access to files generated during container use and, depending on the selected settings, enables persistent data storage on local hard drives. Persistent data storage directly into the NAS or server (i.e., outside of Docker) simplifies backup and disaster recovery procedures, which should be considered part of maintaining this system. Second, while the apps are fully functional within a local network, additional steps may be required for remote access. These steps include securing relevant domains, configuring wildcard certificates, enabling HTTP/2 and HTTP compression, setting up reverse proxies, and forwarding specific ports on the NAS/server as part of router configuration. These measures are essential for users or guests who wish to access, for instance, Mattermost or Nextcloud from outside their local networks (e.g., outside of a university campus).

### Hardware and operating systems (OS)

Although during our learning process we used proprietary hardware and associated OS, we highly encourage new users to explore the use of alternative ways to deploy their own LabOps under more FOSS-friendly environments. To deploy our LabOps solution, we used a Synology NAS (model DS220+, later migrated to DS723+). Both systems performed well for approximately 10 simultaneous users, providing continuous access to around approximately 1 TB of data, while supporting messaging and collaborative writing. Although deploying this system using a Synology NAS is relatively simple—given the extensive documentation and intuitive, non-command-line–dependent options for installing and maintaining services—Synology’s operating system remains part of a closed environment. Synology and similar companies offer user-friendly, largely stable solutions for deploying and serving these systems out of the box. Compared to custom-built NAS setups, these preconfigured systems—often based on modified FOSS (e.g., Synology’s DiskStation Manager is Linux-based)—oftentimes offer reduced security concerns and increased convenience (both in software and hardware) for first-time users. The system presented in this paper can also be deployed on servers (instead of NAS) or even using less specialized hardware (e.g., laptop, a mini computer) and non-proprietary operating systems such as TrueNAS or Unraid. In the latter case, there are long-term benefits, including enhanced customization options for both software and hardware, as well as additional flexibility for users to explore and integrate new features.

## Case study

There are numerous situations in which a FOSS workflow can support state-of-the-art research and enhance collaboration both within and across disciplines. Below, we briefly outline three scenarios: small-scale collaboration on a paper, collaborative grant writing, and interdisciplinary research efforts. A faculty member (F1) is collaborating on a grant proposal with a member of their lab (S1) and a faculty member at another institution (F2). To simplify communication among all three parties (F1, F2, S1), a channel is first created in Mattermost, including all members. Next, relevant files (e.g., a call for proposals, notes from previous conversations) are stored in a new folder in Nextcloud. A README.md file is added to the same folder to provide a brief description and include additional resources, enabling users to quickly understand the contents upon opening it. In this scenario, only the PI and their student (F1 and S1) have accounts in Nextcloud. Since the second faculty member (F2) is only a short-term contributor, creating an account and explaining the folder structure is probably unnecessary. Instead, F2 is granted access via a public link to the Nextcloud folder with edit privileges, allowing them to modify any files within it. Inside Nextcloud, F1 creates the first draft of the proposal as a DOCX file using OnlyOffice. F1 tags S1 in sections where input is required. Both users can simultaneously open and edit the same version of the document, making real-time changes and adjusting the narrative as needed. All edits are saved directly to Nextcloud. The versioning system integrated between Nextcloud and OnlyOffice allows the team to view and restore previous versions of the document as needed. F2 can access the most recent version of the proposal through the shared Nextcloud link, make changes or leave suggestions in OnlyOffice, and provide additional feedback via Mattermost. The folder link can also be pinned in the Mattermost chat for quick access. Tasks can be distributed among team members using Boards, also available in Mattermost. S1, who is in charge of managing references, uses Zotero to collect citations and insert them into the document via Zotero’s plugin in OnlyOffice. Meetings are scheduled using the Nextcloud Calendar or Radicale, and some meetings are held through Nextcloud Talk. Finally, suppose the student is responsible for generating multiple figures in R that need to be updated as new data becomes available. To support this workflow, the student creates a repository using GitHub (not self-hostable), GitLab, or Gitea (a FOSS alternative discussed above). Code changes are tracked within these platforms, ensuring version control and reproducibility. Links to the relevant repository are also added to Mattermost and the Nextcloud README.md for simplified access and streamlined collaboration. This represents and ideal scenario of collaboration within this LabOps framework. However, as discussed under “Strategies for adoption”, labs can choose to use a fraction of these tools as they see fit. Just as FOSS tools support collaboration in research, they can also play a transversal role in grant writing (e.g., a shared Nextcloud folder with edit access for contributors, paired with collaborative writing in OnlyOffice) and interdisciplinary collaboration (e.g., Mattermost channels connect teams across institutions, while Nextcloud centralizes datasets, protocols, and editable documents).

## Integration with other research-related workflows

The set of tools organized as a workflow in this paper can also be tailored to the specific needs of individual labs. The workflow presented here extends beyond file sharing, collaborative document editing, and calendar sharing capabilities. For instance, Git/GitHub for version-controlled research collaboration is integral to many academic workflows. These integrations align with widely adopted practices in reproducible computational research, including the use of Jupyter Notebooks and Quarto. In some cases, Gitea or GitLab can serve as FOSS alternatives for managing code-based collaboration around projects. Self-hosted services can also provide access to programming environments through platforms such as JupyterHub (or JupyterLab) and Code Server. Similarly, manuscript writing can be supported through self-hosted versions of Overleaf or Etherpad. Labs often rely on tools for taking and organizing notes (e.g., HedgeDoc, Obsidian) and for documentation. In those cases, FOSS platforms like Logseq, WikiDocs, and Docmost can help lab members stay organized, support their workflows, and enable community-oriented contributions to shared documents.

## Strategies for adoption

Immediate adoption of this LabOps workflow—or similar self-hosted systems—is likely not ideal for all teams, particularly due to the upfront hardware costs and the complexity of securely deploying the system. Migration procedures and the stabilization of services could also slow down productivity if all components are moved at once. A more practical approach to adopting FOSS alternatives may involve incremental or hybrid strategies. This approach allows teams to progressively integrate FOSS tools rather than transitioning entirely to a self-hosted environment from the outset. A phased strategy could (1) reduce barriers to entry while enabling users to assess and adapt to new tools over time, and (2) allow for the implementation of user feedback relative to existing proprietary alternatives focused on specific tasks (e.g., collaborative writing). To our knowledge, multiple institutions—primarily located in Europe—are hosting FOSS alternatives at the institutional level (e.g., Nextcloud, Mattermost, OnlyOffice), enabling community members to explore FOSS software as viable alternatives to proprietary solutions.

Sequential adoption can also help ensure compatibility across devices and operating systems—including mobile, macOS, and Linux—which is essential for broader adoption. In fact, due to built-in redundancy across self-hosted applications, along with the expected modularity, our workflow is well-suited to support phased implementation. This partial or stepwise approach allows teams to adopt only the components that align with their current needs and expand the system gradually as their capacity grows.

We also highlight that the adoption of this workflow strongly depends on engagement with potential users, stakeholders, among other members of different academic institutions and networks. Although our goal does not target individual labs to deploy their own self-hosted FOSS systems (see “Conclusion”), collaboration around the LabOps workflow presented in this paper is highly encouraged through software suggestions, new issue requests, pull requests, among others (See Tip #6 in ref. [20]). This approach will ensure that a LabOps v.1 will be constantly reviewed, and eventually, an enhanced LabOps v.2 will eventually be released.

Finally, we mentioned that this workflow depends on a hardware component. One barrier to adoption is hardware availability. Hardware is expensive—especially when underutilized—and requires an upfront investment that only makes sense if the FOSS infrastructure is used in the long term. In some cases, cloud-based FOSS hosting services (generally available via subscription) are offered for some of the tools included in our workflow. These services, available as short-term trials or demos, can support internal discussions about the relevance and usability of a given tool without requiring upfront hardware purchases. Likewise, cloud-based solutions may be particularly valuable for labs that are interested in using the software but are either unwilling or unable to manage physical infrastructure on their own.

## Challenges in FOSS and proprietary solutions

Commonly used alternatives to the FOSS workflow we present include cloud storage services like OneDrive, Google Drive, and Dropbox. These services allow clients to store data under specific conditions and restrictions. Popular communication tools such as Microsoft Teams, Slack Discord and WhatsApp, as well as calendar administration systems like Google Calendar and Microsoft Outlook, are widely adopted by research teams. EndNote is another popular choice for citation management. While these tools are powerful and address many of the needs of research teams, they fall short in several critical areas important for research environments [21,22]. Accessibility is often limited, as most of this software requires purchasing a license or subscription. Vendor lock-in, price control strategies through subscriptions, and changes in terms and conditions are common issues under this model. Customization options—such as added functionality, data retention, and storage capacity—are typically only available through commercial upgrades. Inherent privacy and security concerns regarding how these companies manage or access data stored on their servers also remain among the key drawbacks of proprietary and closed-source solutions.

While FOSS alternatives offer many advantages, self-hosting these products also comes with notable challenges. Systems designed for collaboration often require additional hardware for deployment and a certain level of technical expertise for ongoing maintenance. These requirements can be especially restrictive for small teams, labs with limited computational experience, or those operating under funding constraints. In many cases, support for deploying, using, maintaining, and further developing FOSS is obtained through user communities associated with specific tools. Most of the software discussed in our LabOps workflow is relatively active at the time of publication (e.g., community blogs, GitHub issue tracking). However, this does not guarantee that a project referenced here will not become stale or deprecated in the future. If a key tool loses active development, labs may face difficulties related to maintenance, updates, and compatibility.

In contrast, proprietary software is generally easier to install and presents fewer initial barriers to access and use. Maintenance tends to be more consistent, and financial incentives often ensure that updates are released on a regular basis. That said, self-hosted environments offer important benefits, particularly in terms of flexibility and control. Labs can customize and scale their hardware and storage to meet specific needs. The LabOps system presented here is designed primarily to support collaboration on small to medium-sized files (approximately 1 TB of data is consistently available to all members in our deployment). It is not intended to replace long-term or archival storage, though such solutions can be integrated depending on the needs of the collaboration. Data-intensive laboratories may benefit from self-hosting software on servers that can be upgraded and tailored over time. Still, purchasing and managing hardware requires a significant upfront investment relative to license or subscription models and adds an additional layer of maintenance. However, these initial costs could lead to long-term savings depending on system use, scalability, and intended goals.

Self-hosted solutions also give users direct control over their data by limiting its flow to local and trusted servers. The open-source nature of FOSS allows users to inspect code for security vulnerabilities or unwanted tracking—enhancing both privacy and overall workflow security. Given that research environments often involve sensitive data (e.g., personal, health-related, or novel research), self-hosting—when implemented effectively—can significantly strengthen workflows by directly addressing these concerns. Security, however, remains one of the most prominent challenges of self-hosting. Risks such as data breaches and data loss must be carefully considered, especially when exposing services to the internet. Hosting systems within university networks offers the advantage of leveraging existing institutional security infrastructure, rather than building one independently. However, this added layer of protection may come with limitations, as universities often restrict access to specific ports or applications for users outside the campus network or not connected via VPN.

FOSS workflows remain relatively rare in academia, and several factors contribute to this. First, there is a steep learning curve associated with setting up and maintaining these systems. Universities often lack incentives to adopt them, as long-term contracts are typically established with major providers like Google and Microsoft. Additionally, the process of deploying and maintaining self-hosted systems tends to require more hands-on involvement than existing commercial services, which can deter adoption. Furthermore, managing data in decentralized NAS setups is sometimes not ideal for academic institutions or funders, who may prefer centralized and professionally managed solutions. Security concerns related to data access and protection further discourage widespread FOSS adoption in academic environments. Lastly, the upfront investment required to implement self-hosted systems contrasts with the smaller, recurring costs associated with subscription-based services.

Despite these challenges, it is important to note that comprehensive documentation and robust community support are widely available to help users troubleshoot and optimize FOSS tools. These resources can alleviate many of the difficulties encountered during setup and maintenance, offering a pathway for broader adoption over time.

## Conclusions

FOSS-based workflows offer a compelling alternative to proprietary collaboration tools. The workflow we present in this paper provides similar—or even enhanced—functionality without the costs associated with subscription-based solutions. That said, hardware is still required for deployment (e.g., NAS, server). While challenges related to deployment, maintenance, and security do exist, comprehensive community support and detailed documentation make FOSS a viable option for academic teams. A significant advantage of FOSS is the opportunity for customization and scalability, enabling research teams to maintain full control over their digital infrastructure, balance costs, and improve security and privacy. Although the workflow presented here includes a predefined set of tools, it remains highly customizable. For instance, it could be further enhanced by integrating note-taking or programming-related tools, refining user interfaces, or incorporating advanced security features to protect sensitive research data. Adopting FOSS in academia could reduce dependency on commercial software while enabling long-term cost savings and greater independence. We discuss strategies for adoption and suggest that a modular or hybrid approach—in which FOSS tools replace existing services incrementally—may be an optimal path for supporting a smooth transition.

Although we present a workflow for self-hosted collaboration in academic environments, this paper is not a call for every lab to deploy such systems. Widespread deployment of these workflows could be inefficient in many contexts and may negatively affect productivity in other key areas. Scientists should not be expected to act as full IT departments. In fact, we suggest that “LabOps” should be viewed as an alternative approach to communication and collaboration for research labs—not necessarily a workflow that must be deployed independently by each lab. While some labs may have staff members capable of supporting these systems for internal use, we acknowledge that, for many research teams, the LabOps workflow may be largely inaccessible or impractical to implement. This can be attributed to several factors, including (1) financial and personnel constraints, (2) limited access to software or technical infrastructure, and (3) the convenience and familiarity of existing tools. Recognizing that viable alternatives to closed-license solutions exist is an important first step in fostering productive discussions about the balance between FOSS and other collaboration frameworks.

Our goal is to demonstrate that viable, privacy-friendly, and user-focused alternatives exist to the typical software toolkits provided by institutions. Broader awareness of available alternatives may influence how academic units and institutions approach spending and infrastructure decisions. Similarly, our paper does not advocate for proprietary hardware solutions, which—despite offering convenience for self-hosting—ultimately contrast with the broader goals of the FOSS framework. While our own deployment makes use of NAS, we encourage readers to consider hardware built for executing services rather than simply for data storage. Finally, in an era of AI-powered assistants, we acknowledge that simple technical questions—such as understanding the structure of folders or the meaning of configuration parameters—could increasingly be answered through large language models (LLMs). However, one of the key advantages of community-developed and community-supported systems, especially those that remain active, is the ability to receive support directly from other users—not from bots or scripted customer service. Help is often found in blogs, support forums, GitHub repositories, and other community-driven spaces.

## Abbreviations

FOSS: free and open source software
LabOps: laboratory operations
LLMs: large language models
NAS: network attached storage
OSI: open source initiative

## References

[1] Sonnenwald DH. Scientific collaboration. Annu Rev Inf Sci Technol. 2007;41(1):643–81.

[2] Ravankar A, Imai S, Ravankar A. Managing the Project: The Essential Need for Project Management Training and Education in Graduate Schools. In: 2019 8th International Congress on Advanced Applied Informatics (IIAI-AAI), 2019. 420–5. doi: 10.1109/iiai-aai.2019.00092

[3] Dankoski M, Palmer M, Banks J, Brutkiewicz R, Walvoord E, Hoffmann-Longtin K, et al. Academic writing: Supporting faculty in a critical competency for success. J Fac Dev. 2012;26(2):47–54.

[4] Chase J-AD, Topp R, Smith CE, Cohen MZ, Fahrenwald N, Zerwic JJ, et al. Time management strategies for research productivity. West J Nurs Res. 2013;35(2):155–76. doi: 10.1177/0193945912451163 . 22868990

[5] Wouters L, Creff S, Bella EE, Koudri A. Collaborative systems engineering: Issues & challenges. In: 2017 IEEE 21st International Conference on Computer Supported Cooperative Work in Design (CSCWD), 2017. 486–91.

[6] Nosek JT, McManus M. Collaboration Challenges: Bridging the IT Support Gap. Inf Syst Manag. 2008;25(1):3–7. doi: 10.1080/10580530701777081

[7] Soveizi N, Turkmen F, Karastoyanova D. Security and privacy concerns in cloud-based scientific and business workflows: A systematic review. Future Gener Comp Syst. 2023;148:184–200. doi: 10.1016/j.future.2023.05.015

[8] Pastore S. Social networks, collaboration and groupware software for the scientific research process in the web 2.0 world. In: Proceedings of the 7th WSEAS International Conference on Artificial intelligence, knowledge engineering and data bases 2008;403–408.

[9] David S. Perspectives on Free and Open Source Software. Feller J, Fitzgerald B, Hissam SA, Lakhani KR. Cambridge, MA: The MIT Press.

[10] Dasgupta J. Imparting hands-on industry 4.0 education at low cost using open source tools and python eco-system. New Paradigm of Industry 4.0: Internet of Things, Big Data & Cyber Physical Systems. Cham: Springer International Publishing. 2019;37–47.

[11] Androutsellis-Theotokis S, Spinellis D, Kechagia M, Gousios G. Open Source Software: A Survey from 10,000 Feet. Foundations and Trends in Technology, Information and Operations Management. 2011;4(3–4):187–347.

[12] Perens B. The open source definition. Open sources: voices from the open source revolution. 1999;171–88.

[13] Fortunato L, Galassi M. The case for free and open source software in research and scholarship. Philos Trans A Math Phys Eng Sci. 2021;379(2197):20200079. doi: 10.1098/rsta.2020.0079 . 33775148

[14] Maracke C. Free and Open Source Software and FRAND‐based Patent Licenses: How to Mediate Between Standard Essential Patent and Free and Open Source Software. J World Intellect Prop. 2019;22(3–4):78–102.

[15] Weber S. The Success of Open Source. Harvard University Press; 2004 Dec 31.

[16] Napoleao BM, Petrillo F, Halle S. Open Source Software Development Process: A Systematic Review. In: 2020 IEEE 24th International Enterprise Distributed Object Computing Conference (EDOC), 2020. 135–44. doi: 10.1109/edoc49727.2020.00025

[17] Levine SS, Prietula MJ. Open collaboration for innovation: Principles and performance. Organ Sci. 2014;25(5):1414–33. doi: 10.1287/orsc.2013.0872

[18] Wulf WA. The collaboratory opportunity. Science. 1993;261(5123):854–5. doi: 10.1126/science.8346438 . 8346438

[19] Chin G Jr, Lansing CS. Capturing and supporting contexts for scientific data sharing via the biological sciences collaboratory. In: Proceedings of the 2004 ACM conference on Computer supported cooperative work, 2004. 409–18. doi: 10.1145/1031607.1031677

[20] Littauer R, Wilson G, Ainali J, AlOmar EA, Arabas S, Saibene YB, et al. 10 quick tips for making your software outlive your job. arXiv. 2025. doi: 2505.06484

[21] Nederbragt AJ. On the middle ground between open source and commercial software – the case of the Newbler program. Genome Biol. 2014;15(4):113. doi: 10.1186/gb4173 . 25180324 PMC4054848

[22] Lees JM. Open and free: Software and scientific reproducibility. Seismol Res Lett. 2012;83(5):751–2.

